# Novel Modification of Potassium Chloride Induced Cardiac Arrest Model for Aged Mice

**DOI:** 10.14336/AD.2017.0221

**Published:** 2018-02-01

**Authors:** Huaqin Liu, Zhui Yu, Ying Li, Bin Xu, Baihui Yan, Wulf Paschen, David S Warner, Wei Yang, Huaxin Sheng

**Affiliations:** ^1^The Multidisciplinary Neuroprotection Laboratories, Department of Anesthesiology, Duke University Medical Center, Durham, NC, USA; ^2^Department of Anesthesiology, The 4^th^ Hospital of Hebei Medical University, Shijiazhuang, China; ^3^Department of Critical Care Medicine, Renmin Hospital of Wuhan University, Wuhan, China; ^4^Department of Cardiology, The 5^th^ Hospital of Tianjin, Tianjin, China; ^5^Department of Environmental Health, China Medical University, Shenyang, China; ^6^Department of Anesthesiology, The Second Affiliated Hospital of Xi’an Jiaotong University, Xi’an, China

**Keywords:** cardiac arrest, resuscitation, mouse model, aging

## Abstract

Experimental cardiac arrest (CA) in aging research is infrequently studied in part due to the limitation of animal models. We aimed to develop an easily performed mouse CA model to meet this need. A standard mouse KCl-induced CA model using chest compressions and intravenous epinephrine for resuscitation was modified by blood withdrawal prior to CA onset, so as to decrease the requisite KCl dose to induce CA by decreasing the circulating blood volume. The modification was then compared to the standard model in young adult mice subjected to 8 min CA. 22-month old mice were then subjected to 8 min CA, resuscitated, and compared to young adult mice. Post-CA functional recovery was evaluated by measuring spontaneous locomotor activity pre-injury, and on post-CA days 1, 2, and 3. Neurological score and brain histology were examined on day 3. Brain elF2α phosphorylation levels were measured at 1 h to verify tissue stress. Compared to the standard model, the modification decreased cardiopulmonary resuscitation duration and increased 3-day survival in young mice. For aged mice, survival was 100 % at 24 h and 54% at 72 h. Neurological deficit was present 3 days post-CA, although more severe versus young mice. Mild neuronal necrosis was present in the cortex and hippocampus. The modified model markedly induced elF2α phosphorylation in both age groups. This modified procedure makes the CA model feasible in aged mice and provides a practical platform for understanding injury mechanisms and developing therapeutics for elderly patients.

The American Heart Association reports that there are >350,000 cases of cardiac arrest (CA) per year in the United States, accounting for 50% of all cardiovascular deaths [1]. CA is a major cause of death in the elderly and the incidence increases with age [2].

Clinical investigations recently analyzed factors associated with outcome and long-term survival in elderly patients with out-of-hospital CA [3-6], as well as the effect of age on survival and outcome [7, 8]. Although the incidence of favorable CA neurological outcomes has improved, the resuscitation success rate remains low [3]. Cardiopulmonary resuscitation duration was reported to be independently associated with favorable functional status at hospital discharge [9]. There is need to advance therapy to improve clinical outcome.

Most experimental studies focusing on therapeutic development for CA have used young healthy animals [10-13]. Aged animals are rarely investigated, but aging likely poses different responses to CA [14-16]. Compared to 3-6-month-old rats, overall survival rate, hypoxic ventilatory response, and brain mitochondrial respiratory function were decreased in 24-month old rats following CA and resuscitation, while hippocampal CA1 injury was increased [15]. The Stroke Therapy and Industry Roundtable recommends that preclinical research should be performed in aged animal models to expand clinical relevance [17]. A major factor discouraging investigators from using aged animal models is poor post-CA survival [18]. Ikeda et al. [16] reported 100% mortality at 24 hours with 8 min CA and 21% mortality with 7 min CA in 78-87-week-old mice. Xu et al. [14] reported a 50% mortality at 4 days post-CA in 6-month-old rats and 0% survival in 24-month-old rats. The purpose of this study was to develop an improved CA model in aged mice to address this limitation.

## MATERIAL AND METHODS

This study was approved by the Duke University Animal Care and Use Committee, and complied with the Guide for the Care and Use of Animals published by the National Institutes of Health. 8-10-week old and 22-month old male C57Bl/6 mice were purchased from Jackson Laboratory and the National Institute on Aging. All mice were held in the Duke University vivarium under standard housing conditions for one week prior to surgery.

### Modified cardiac arrest and cardiopulmonary resuscitation model

**Figure 1.**
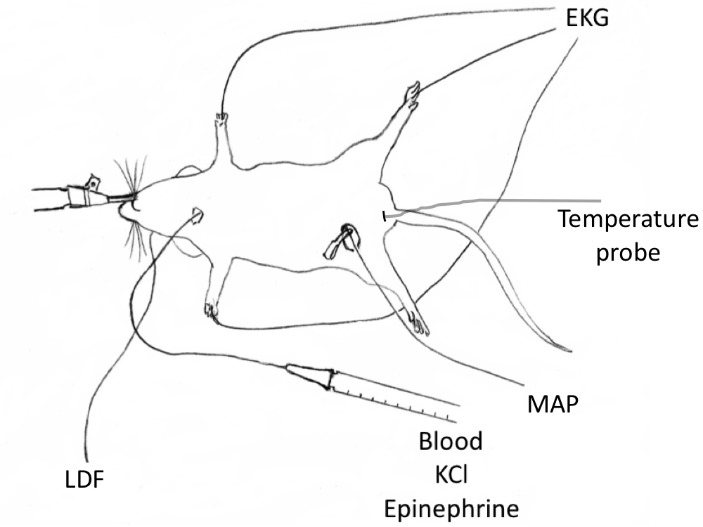
Diagram of the modified mouse cardiac arrest model The difference from other models is that blood was withdrawn from jugular vein before onset of cardiac arrest to enable use of a small amount of KCl (30 µl 0.5 M) to induce cardiac arrest. Blood was re-infused 3 minutes following cardiac arrest onset. LDF = laser Doppler flow, MAP = mean arterial blood pressure and EKG = electrocardiography.

Mice were anesthetized with 5% isoflurane in 40% oxygen balanced with nitrogen, and endotracheally intubated with a 20-gauge IV catheter (BD Insyte^TM^) (Fig. 1). The lungs were mechanically ventilated with the tidal volume of 0.7 ml and the rate of 105 strokes per min (Rodent ventilator model 683, Harvard). Isoflurane was decreased to 1.8% during surgery. Using surface heating/cooling, rectal temperature was maintained at 37± 0.2°C before CA and then only monitored during CA, cardiopulmonary resuscitation (CPR), and post-CA recovery. Needle electrodes were inserted into both forelegs and the left hind leg respectively to record the electrocardiogram (EKG). Neck area was shaved and prepared. A 1 cm skin incision was cut and tight jugular vein was exposed. A PE-10 catheter was placed in the right jugular vein for blood withdrawal and fluid infusion. After 15 min for physiologic stabilization, 300 µl of blood were withdrawn into a 1 ml syringe containing 5 units of heparin in 100 µl normal saline. Then a 50 µl Hamilton syringe was connected to the PE-10 catheter immediately after blood withdrawal and 30 µl 0.5 M KCl was rapidly infused into the jugular vein. CA was confirmed by loss of EKG activity. Isoflurane was discontinued. Mechanical ventilation and surface heating ceased. A timer was started. The withdrawn blood was reconnected to the PE-10 catheter and slowly re-infused intravenously beginning 3 min after asystole onset. Then, a second syringe was connected to the PE-10 catheter for epinephrine infusion. At 7.5 min, delivered oxygen was adjusted to 100% and epinephrine (32 µg/ml) was infused at 20 µl/min. At 8 min, mechanical ventilation was resumed and an IV bolus of 3.2 µg epinephrine (100 µl) was rapidly given followed by a continuous slow infusion. Chest compression was performed using a single finger at 300 stroke/min until an EKG sinus rhythm appeared. The epinephrine infusion was then stopped. The maximal epinephrine infusate volume was restricted to < 300 µl. The venous catheter was removed. The skin incision was closed with suture and covered with triple antibiotic ointment. Mice were disconnected from the ventilator and the trachea was extubated when spontaneous ventilation was adequate. Mice were administered 50% oxygen in the chamber overnight before being returned to their home cage.

### Experiment 1: Compare the modified model with the traditional model in young adult mice.

Male C57Bl/6 mice (8-10 weeks of age) were subjected to 8-min CA and CPR. Mice were randomly assigned to the respective models using a scientific software in www.graphpad.com. The volume of KCl solution used to induce CA was 30 µl in the modified model (n=5) and 50 µl in the traditional model (n=5) [16]. Blood withdrawal was not performed in the traditional model. Resuscitation time represented the duration from the beginning of onset of chest compressions until the onset of normal sinus rhythm. Resuscitation time was recorded by an observer who was blind to group assignment. Mice were allowed to survive 72 h and mortality was measured.

An additional 5 mice were subjected to the modified model (Fig. 1). An arterial catheter was placed in the femoral artery to measure mean arterial blood pressure (MAP). A laser Doppler flow (LDF) probe was glued on the right temporal bone. LDF was continuously monitored (VMS-LDF-1, Moor Instruments). A baseline reading, taken 5 min prior to CA onset, was considered 100%. MAP, LDF, and rectal temperature were recorded 5 min prior to CA, 4 and 8 min after CA onset, and 5, 10, 20 and 30 min post-CA. These animals were then euthanized.

### Experiment 2. Cardiac arrest and cardiopulmonary resuscitation in aged versus young adult mice.

Aged C57Bl/6 mice (22-months of age) were subjected to 8 min CA/CPR using the modified procedure (n=10). Young adult male C57Bl/6 (8-10 weeks of age) mice were contemporaneously subjected to the same procedure (n=9). The resuscitation time was recorded as described in Experiment 1. All mice were allowed to survive 72 h. Post-CA functional recovery was monitored daily by measurement of spontaneous locomotor activity. Mice were placed in a SmartCage^TM^ (AfaSci Research Laboratories, Redwood City, CA) and allowed to freely move for 6 min. Parameters including activity time, travel velocity, and travel distance were automatically detected and calculated over a 5-min interval beginning 15 secs after placement in the apparatus. Five naïve mice were also examined and served as a normal reference for spontaneous activity.

At 72 h post-CA, an established neurologic examination designed to detect motor deficits in the rat [19] and modified for the mouse [20, 21] was performed. Mice were placed on a horizontal 10 x 20 cm screen (grid size 0.2 x 0.2 cm) that could be rotated to 90º (vertical). The duration that the mouse was able to adhere to the vertical screen was recorded to a maximum of 15 sec (allowing a total of three points). Next, the mouse was placed at the center of a horizontal wooden rod (1.5 cm diameter), and the time that the mouse was able to remain balanced on the rod was recorded to a maximum of 30 sec (allowing a total of 3 points). Finally, a prehensile traction test was administered. The time that the mouse was able to cling to a horizontal rope was recorded to a maximum of 5 sec. From these three tests, a total motor score (9 possible points) was computed.

Mice were then anesthetized and intravascularly perfused with heparinized normal saline followed by 10% formalin. Paraffin-embedded brain sections were serially cut (5µm thick) and stained with acid fuchsin/celestine blue. With the investigator using light microscopy and blinded to age groups, necrotic neurons in the CA1 sector of the hippocampus and neocortex were counted at standardized intervals.

An additional experiment was performed to examine post-CA neurological deficit and histological damage with pericranial temperature controlled at 37ºC during CA and for 30 min post-CA/CPR. Aged mice were subjected to 8 min CA (n=5) or sham surgery (n=5) as described above. Spontaneous activity and brain histologic outcome were measured at 72 h, as described above.

### Experiment 3. Determine endoplasmic reticulum (ER) stress from the modified CA.

Young adult (8-10 weeks) and aged (22 months) male C57Bl/6 mice were subjected to 8 min of CA/CPR or sham surgery (n=3 per group). Brain tissue was harvested at 1 h post-CA. Phospho-eukaryotic translation initiation factor 2α (p-elF2α) western blot analysis was performed as described previously [22]. Brain cortex samples were homogenized with sonication using 2% SDS lysis buffer. Protein samples were separated on pre-cast SDS-PAGE gels (Bio-Rad) and were transferred to PVDF membranes. Membranes were blocked with TBST containing 5% BSA, and incubated with a primary antibody of p-elF2α (1:500, cell signaling) overnight at 4^o^C. After extensive washing, membranes were incubated with a secondary antibody for 1 h at room temperature. Proteins were then visualized using an enhanced chemiluminescence analysis system (GE Healthcare). After exposure, membranes were stripped and re-probed for β-actin as loading control. Image analysis was performed using the Image J program.

### Statistical Analysis

All data were confirmed with normal distribution. Resuscitation time was compared using the unpaired student T-test and are expressed as mean ± SD. Spontaneous activity measures were compared using repeated measures analysis of variance. Neurological scores were analyzed using the Mann-Whitney U statistic and are expressed as median ± interquartile range. Survival rates were analyzed with the Mantel-Cox test and are presented as percentages. Significance was assumed if *P* < 0.05.

**Figure 2.**
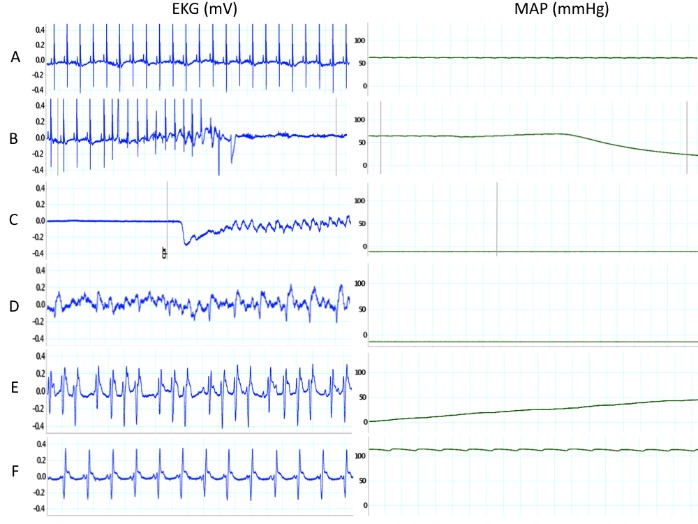
Electrocardiography (EKG) and mean arterial blood pressure (MAP) changes in cardiac arrest (**A**) pre-injury phase, (**B**) cardiac arrest onset, (**C**) initial resuscitation phase (**D**) resuscitation phase with absent blood pressure, (**E**) late resuscitation phase, (**F**) recovery phase following resuscitation. For the x-axis in A-F, each square = 0.25 sec.

## RESULTS

### Experiment 1: The model modification shortened resuscitation duration and improved post-CA survival in young adult mice.

**Figure 3.**
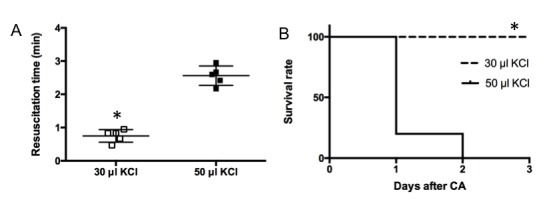
Resuscitation time and % survival over 72 h in young adult cardiac arrest mice subjected to 30 µl- or 50 µl KCl-induced cardiac arrest (n=5/group) (**A**) resuscitation time; (**B**) Survival rate. Squares represent individual mice. Horizontal bars represent mean±SD, * P<0.05 vs. 50 µl KCl group.

All mice were successfully resuscitated from 8 min CA. A typical pattern of EKG and MAP changes during the procedure is shown in Fig. 2. By decreasing KCl from 50 µl to 30 µl, the time from the beginning of resuscitation to restoration of EKG sinus rhythm was decreased to 0.75 ± 0.18 min in the modified model from 2.56 ± 0.29 min in the traditional model (p= 0.014, Fig. 3A). All 30 µl KCl-induced CA mice survived 72 h. In the 50 µl KCl-induced CA group, mortality was 100% by 48 h (p < 0.05, Fig. 3B).

**Table 1:** Physiologic values during cardiac arrest and resuscitation.

	Rectal Temperature (°C)	Mean Arterial Pressure (mmHg)	LDF (% baseline)
Pre CA-4 CA-8 R-5 R-10 R-20 R-30	37.3 ± 0.2 36.7 ± 0.4 35.9 ± 0.5 34.8 ± 0.5 34.4 ± 0.5 33.3 ± 0.7 32.5 ± 0.6	78 ± 2 7 ± 1 6 ± 1 123 ± 22 58 ± 12 66 ± 16 82 ± 14	100 2 ± 1 2 ± 0 64 ± 8 54 ± 12 77 ± 21 87 ± 38

Pre = before cardiac arrest (CA); CA-4 and CA-8 = 4 and 8 min after CA onset; R-5, 10, 20 and 30 = 5, 10, 20 and 30 min after initiation of cardiopulmonary resuscitation, respectively. LDF = laser Doppler flow.

Rectal temperature, MAP and LDF values are depicted in Table 1. Most notably, hypothermia was present post-CA.

### Experiment 2: The modified CA procedure is practical for aged mice

**Figure 4.**
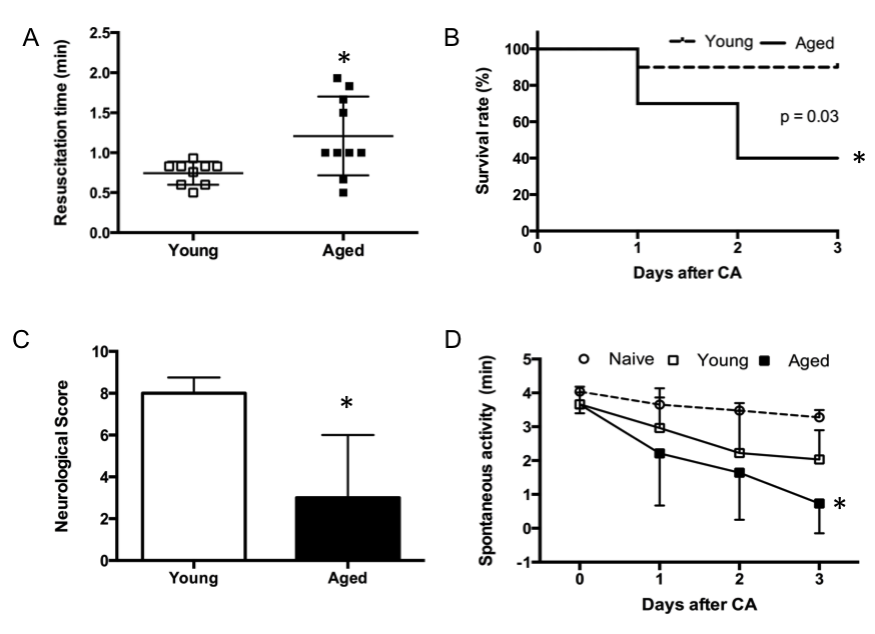
Resuscitation duration, functional recovery and survival curve in aged cardiac arrest mice (n=9-10/group) (**A**) resuscitation time, (**B**) survival curve, (**C**) neurological score, (**D**) spontaneous motor activity. * *P*<0.05 vs. young adult group.

**Figure 5.**
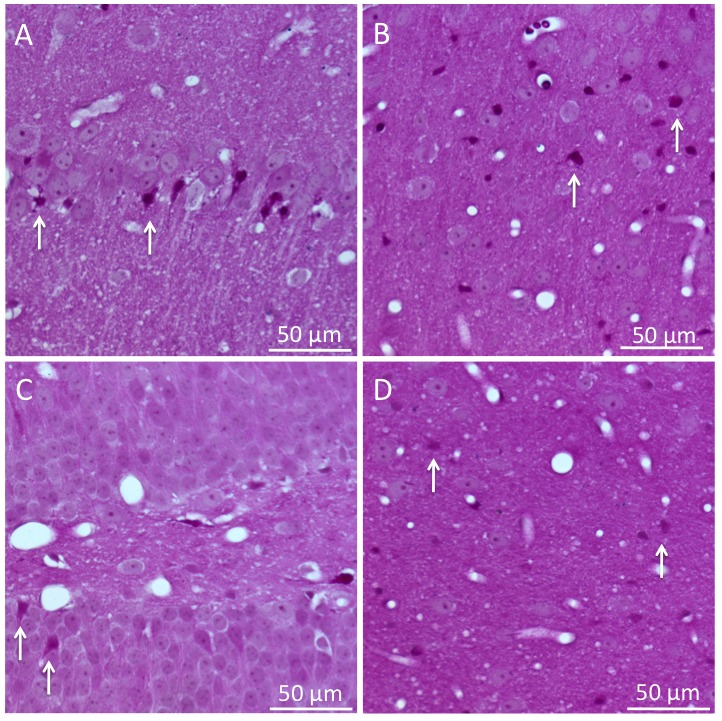
Representative brain histology 3 days post-cardiac arrest (**A**) hippocampal CA1, (**B**) cortex, (**C**) dentate gyrus, (**D**) thalamus. Arrows point to dead neurons.

All aged mice were resuscitated successfully although the time taken was longer than for young adults (aged = 1.21 ± 0.49 min, young adults = 0.74 ± 0.14 min, p= 0.014, Fig. 4A). The mice were quiescent following CA/CPR, but recovered ambulatory activity by the following day. There was a clear effect of age on survival and neurologic morbidity (Fig. 4B, p= 0.03). In the aged mice, the survival rate was 100% at 24 h post-CA, but decreased to 40% at 72 h. In contrast, 90% of the young adults survived 72 h. Neurological score at 72 h was 3 ± 3 in surviving aged rats and 8 ± 3 in young adults (Fig. 4C, p= 0.03). Spontaneous locomotor activity gradually declined over the 3-day recovery interval and was worse in the aged versus young adult mice (Fig. 4D, *p*= 0.04 in activity time). Travel distance declined as well (*p*< 0.01). No difference was present in velocity (p= 0.18). Neuronal necrosis was found in multiple areas including cortex, CA1, dentate gyrus, and thalamus (Fig. 5). There was no intra-group histologic difference in either cortex (aged 8 ± 4 dead neurons/field, young adult 6 ± 3 dead neurons/field, *p*= 0.42) and CA1 (aged 6 ± 3 %, young adult 6 ± 4%, *p*= 0.93).

**Figure 6.**
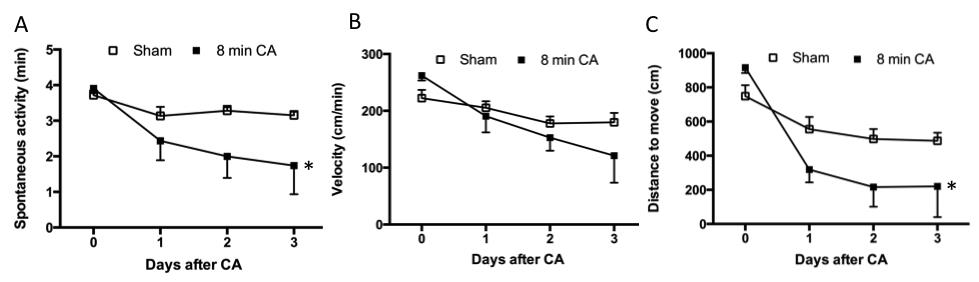
Spontaneous locomotor activity of aged mice subjected to cardiac arrest or sham surgery with rectal temperature controlled at 37ºC until 30 min after resuscitation onset Injured mice had a significant decline of spontaneous activity time (**A**) and travel distance (**C**) compared to sham group (p<0.01). An effect of ischemia on velocity (**B**) was not detected.

Amongst the 5 aged mice for which rectal temperature control was extended to 30 min post-CA, one died at 48 h. The remaining 4 mice survived 72 h post-CA. Three of 4 mice had patchy neuronal death in the cortex; one had sparse neuronal death. Hippocampal CA1 neuronal death in these 4 mice was 9%, 11%, 14% and 78%, respectively. All sham mice survived and had no histologic damage. Spontaneous locomotor activity (*p*<0.01) and distance moved (*p*<0.01) were decreased in injured mice, but there was no effect on travel velocity (*p*= 0.16) (Fig. 6).

### Experiment 3: The modified CA procedure markedly induced ER stress in brain.

**Figure 7.**
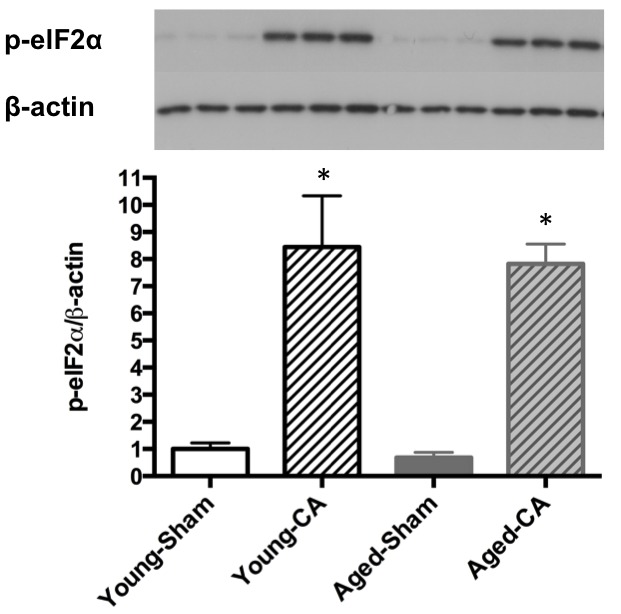
Endoplasmic reticulum stress in brain at 1 h post-CA The level of phosphorylated elF-2α expressed as the ratio of β-actin. * P<0.05 vs. sham group.

In the modified model, phosphorylation of the endoplasmic reticulum stress marker elF2α was dramatically induced at 1 h post-CA in both young and aged mice (Fig. 7, *p*<0.01 versus respective shams). There was no difference between young and aged groups.

## DISCUSSION

There are a variety of CA models available for laboratory study in rodents. Katz et al. [23] pioneered the asphyxial CA model, which has since been emulated by others, where anesthetized rats are subjected to neuromuscular blockade and mechanical ventilation is ceased [24-27]. Hypoxemia leads to a brady-asystolic circulatory arrest within 3-4 minutes following apnea onset. This model is valuable in studying asphyxial cardiac arrest, which demonstrates a different pathophysiologic sequence than sudden onset cardiac arrest [28], which is typically cardiogenic in etiology. Ventricular fibrillation cardiac arrest can be modeled by placing a pacing electrode into the right ventricle to deliver alternating current to the endocardium, resulting in circulatory arrest [29-31]. This procedure, while producing a rapid and uniform time to arrest onset, is surgically complicated and produces thermal injury to the heart.

Perhaps for these reasons, the KCl-induced CA model is frequently employed. The KCl model does not require a device, yet produces near-immediate circulatory arrest. However, the KCl model has proven difficult for routine use in mice because of a high mortality associated with an arrest interval sufficient to cause neuronal injury, even in young mice [32]. The current study demonstrates that a modification of the procedure can substantially improve survival in mice sustaining phosphorylation of elF2α, a marker of severe ER stress, and neuronal necrosis [33]. The circulating blood volume was decreased by blood withdrawal, which allowed a smaller KCl dose to be employed. This is consistent with an increase in plasma potassium concentration sufficient to induce cardiac arrest. Upon reinfusion of the shed blood, plasma potassium concentration becomes diluted. We hypothesized that this method would be associated with a shorter duration of CPR necessary to restore spontaneous circulation. Indeed, CPR time was decreased by almost 2 minutes in young adult mice versus the standard model and held to approximately 73 seconds in aged mice. An abbreviated resuscitation time may offer several advantages. First, mechanical injury to the thorax and its organs is less likely with a shorter interval of chest compression. Second, the interval of complete global ischemia can be more strictly regulated on the basis of experimental requirements. To increase relevance of this work to the human condition, where cardiac arrest occurs most frequently in the elderly, we demonstrated that this model is also applicable to aged mice.

One clinical study in elderly patients reported that the time from collapse to CPR was a major factor affecting outcome [5]. Eight minutes of cardiac arrest is commonly employed in young animal studies [32, 34, 35]. Others used different cardiac arrest durations ranging from 4-10 minutes [15, 16, 36-39], depending on the model, experimental condition, animal species, etc. However, few have reported sufficient recovery in mice beyond 24 hours to allow appropriate assessment of neurologic outcome. In this study, we used 8 minutes of CA for aged mice and the survival rate 3 days post-CA was 40% when temperature was uncontrolled and 80% when temperature was controlled. However, these two experiments were performed by different individuals, which limits our ability to isolate the effect of temperature from individual operator performance, suggesting possibility that operator performance is yet another factor influencing CA outcome in the mouse.

Survival may be further improved if the CA duration is decreased. Important follow-up studies are needed to examine effects of graded CA durations on propensity to survive even longer recovery intervals, consistent with recognized delayed evolution of both histologic and functional recovery from an ischemic insult allowing more valid definition as to whether therapeutic interventions provide sustained benefit. At the same time, it may be reasonable for translational research to accept a model with mortality approaching 50%, as this would provide opportunity for purported therapeutics to improve outcome from a severe insult similar to those typically sustained by humans in out-of-hospital CA.

## Conclusions

This novel method of combining partial exsanguination with a decreased KCl dose to induce CA in both young and aged mice proved useful in decreasing CPR time and mortality over 3 days post-CA. Plausibly, this modification is relevant to other species in which KCl-induced CA is studied. Additional effort is required to extend survival beyond 3 days in a sufficient fraction of mice to allow robust analysis of therapeutic intervention.
